# From animal collective behaviors to swarm robotic cooperation

**DOI:** 10.1093/nsr/nwad040

**Published:** 2023-02-16

**Authors:** Haibin Duan, Mengzhen Huo, Yanming Fan

**Affiliations:** State Key Laboratory of Virtual Reality Technology and Systems, School of Automation Science and Electrical Engineering, Beihang University, Beijing 100083, China; Virtual Reality Fundamental Research Laboratory, Department of Mathematics and Theories, Peng Cheng Laboratory, Shenzhen 518000, China; State Key Laboratory of Virtual Reality Technology and Systems, School of Automation Science and Electrical Engineering, Beihang University, Beijing 100083, China; AVIC Shenyang Aircraft Design and Research Institute, Shenyang 110035, China

**Keywords:** collective behaviors, swarm intelligence, cooperative robotics swarm, human-machine system

## Abstract

The collective behaviors of animals, from schooling fish to packing wolves and flocking birds, display plenty of fascinating phenomena that result from simple interaction rules among individuals. The emergent intelligent properties of the animal collective behaviors, such as self-organization, robustness, adaptability and expansibility, have inspired the design of autonomous unmanned swarm systems. This article reviews several typical natural collective behaviors, introduces the origin and connotation of swarm intelligence, and gives the application case of animal collective behaviors. On this basis, the article focuses on the forefront of progress and bionic achievements of aerial, ground and marine robotics swarms, illustrating the mapping relationship from biological cooperative mechanisms to cooperative unmanned cluster systems. Finally, considering the significance of the coexisting-cooperative-cognitive human-machine system, the key technologies to be solved are given as the reference directions for the subsequent exploration.

## Teaser

Inspired by the fascinating collective behaviors of animals, swarm robotics could be designed to emerge intelligent properties via robotic cooperation for the unmanned swarm system and human-machine system.

## INTRODUCTION

With the rapid technological development of the smart robot, varieties of robots are widely used in modern production and life. Many types of robots have emerged in the swarm, ranging in overall size from macro to micro, even nanometers [1–3]. The intelligent robots with high autonomy could be capable of conducting the sophisticated tasks in complex, unknown environments. A swarm is composed of three or more robots that cooperate to accomplish the tasks with limited or little control from human operators [4]. Like social insects living together in colonies to transcend individual limitations, the swarm-robot system allows for parsimonious solutions to robotic tasks with fewer resources than the comparable single-robot system [5]. This mainly benefits from the simple local interaction rules, which lead to emergent collective behaviors. Inspired by the study of natural systems, engineering principles can be extracted for the application of swarm-robot systems with comparable abilities containing parallel and distributed processing and control, locality of the interaction, scalability of the group, adaptation to the external variation, resilience to the losses and failures of the individual component [6–8]. A swarm-robot system has the following essential attributes [9].

1. Robots are autonomous.
2. Robots can interact with the surroundings and give feedback to modify the environment.
3. Robots possess local perceiving and communicating capabilities.
4. Robots do not exploit centralized swarm control or global knowledge.
5. Robots cooperate with each other to accomplish the given task.

On the basis of these attributes, properties of a swarm-robot system mainly include distribution of the organization, simplicity of the individual, flexibility of the action mode and intelligence on the swarm level, explained as follows [10,11].

### Distribution of the organization

There is no central node in the swarm and each individual follows simple behavioral rules with local perception, planning and communication abilities. Through local information interaction with the environment and neighbors, individuals adjust their behavior modes to adapt to the dynamic environment. A swarm can gain the stigmergy globally. Meanwhile, a distributed framework promotes the self-healing capability and scalability of the system [12]. The introduction or removal of individuals does not require a change in the program or subsequent reprogramming either results in the huge enhancement or reduction of the whole swarm performance. Robust swarm operation in spite of individual failure or environmental changes can be attributed to decentralized control, shared perception information, inherent redundancy from a large-scale swarm and the simplicity of individuals.

### Simplicity of the individual

The abilities or behavior rules adopted by individuals in a group are quite simple. Each individual performs only one or a limited number of actions and makes a few simple responses to external situations. This seemingly clumsy individual behavior makes the group extremely efficient, reflecting the emergence of intelligence. Thus, the swarm system is not a simple sum of individuals, but through the self-organization, coordination and cooperation between individuals, the system realizes multiplication and even exponential growth of the capacity.

### Flexibility of the action mode

Flexibility mainly describes the swarm’s adaptability to the environment. The swarm individuals adapt to environment changes by adjusting their behaviors. In social animals, flexibility is promoted by the redundancy of the swarm size, simplicity of behaviors and other cooperated mechanisms. The flexibility is usually inconsistent with the stability of swarm systems, while the communities in nature tend to be both stable and flexible. From a physical point of view, the swarm could possibly maintain the balance between stability and flexibility during the system phase transition while moving near the critical point [13].

### Intelligence on the swarm level

Individuals exchange and share information by perceiving the environment situation, respond to external stimuli based on certain behavioral rules and enhance the swarm adaptability by adjusting the state, which is a process of learning and evolution. Individuals adaptively change their own behaviors according to feedback information from the environment to achieve strategies and experience learning, thereby obtaining the best adaptability to the external environment. The learning and evolution processes take place both in time and space manifested in the interactive learning of self-historical and external experiences, respectively.

## NATURAL COLLECTIVE BEHAVIOR

The natural collective behaviors of animals, from schooling fish to packing wolves and flocking birds, have long intrigued observers of nature and scientists. The emergent properties have risen in collective motion via simple interactions among individuals, like self-organization, distribution, robustness, adaptivity, etc. Thus, much attention has been placed on revealing delicate local interaction rules, which lead to emergent collective behaviors.

### Bird flocks

Birds often fly in aggregations to display a variety of fascinating phenomena that reflect an obvious degree of swarm coordination and collective response. As a typical example of a small group, pigeon flocks possess a group size from several to dozens of individuals. A well-defined hierarchical structure has been found among pigeon flocks and the birds in the higher rank are more influential in the decision-making process of the flock [14]. From the perspective of evolution, the hierarchical organization of a pigeon flock might be more efficient and stable than an egalitarian one. In contrast, starling flocking is a paradigmatic example of a huge-scale group containing thousands of individuals. The local interactions are ruled by topological (fixed number of neighbors) but not metric distance (neighbors within a certain distance). Furthermore, the fixed number of interaction neighbors is independent of flock density. This interaction mechanism ensures fast information transfer and structural robustness that confer benefits to the collective efficiency [15,16]. Different from the pigeon and starling, larger birds like the ibis show an affinity for V-formation flight, bringing aerodynamic advantages to their annual migration. The following bird in the V-formation could benefit from the aerodynamic up-wash generated by the leading individual [17,18]. Meanwhile, a social dilemma arises around the issue of the volunteers flying in front. A human-guided autumn migrational flight suggested that the ibis cooperate by direct pairwise switches in leading a formation. That is, the time that they spend in the wake of one another is matched with the time spent in the leading position. The direct reciprocation mechanism has a substantial influence on the scale and cohesion of flight formations.

### Fish schools

Fish schools also exhibit complex and coordinated collective behaviors that link individual behavior to the properties of the dynamic swarm [19]. Fish schools move collectively with high alignment through a selfish mechanism [20]. Three pivotal rules exist for the social interaction of fish that are different from other collective animals.

1. Attraction: fish have the ability to copy another individual’s heading to some extent [21].
2. Repulsion: speed regulation is a major component of repulsion, and speed changing is transmitted to the surrounding individuals.
3. Interactions: the single nearest neighbor dominates social interactions to a large extent [22].

These rules help account for the group cohesion and social information amplification responding to rapid variations in speed and direction.

### Wolf packs

Wolf packs regularly engage in cooperative hunting through coordinative actions, which is an important aspect of cooperation [23]. There exists an obvious linear and completely transitive hierarchy based on the direction of submissive behaviors in wolf packs [24]. The rank order is positively correlated to age but not body weight. Dominance relationships remain constant across competitive and not competitive contexts [25]. Moreover, as the more gregarious carnivoran species, wolves have highly developed reconciliation behaviors, which help to reduce aggression between group members, restore social cohesiveness and preserve valuable cooperative relationships [26]. The above relationships help wolf packs to improve the efficiency of cooperative hunting.

### Mapping theory

**Figure 1.**
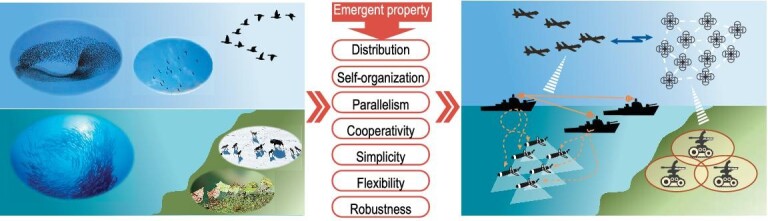
From animal collective behaviors to cooperative unmanned swarm systems.

There are many similarities between the biological swarm and the unmanned system swarm, as listed in Table 1 [10]. The mapping relationships from animal collective behaviors to cooperative unmanned swarm systems are illustrated in Fig. 1. Efforts have been made to model abstract biological collective behaviors mathematically. There are three typical models of collective motion playing foundational roles. Reynolds proposed a distributed behavioral model by imitating the aggregate motion of a flock of birds, namely the boid flock model [27]. Three basic rules in the collective group have been summarized as collision avoidance, velocity matching and flock centering. In order to investigate clustering, transport and phase transition in nonequilibrium systems, a novel pattern is described to explore the appearance of the self-ordered particle motion in the biologically encouraged interaction [28]. In the Vicsek model, the position and the angle of the particle are defined as


(1) \begin{eqnarray*} {{\mathbf {x}}_{i}}( t+1 )={{\mathbf {x}}_{i}}( t )+{{\mathbf {v}}_{i}}( t )\cdot \Delta t, \end{eqnarray*}

(2) \begin{eqnarray*} \theta ( t+1 )={{\langle \theta ( t ) \rangle }_{r}}+\Delta \theta . \end{eqnarray*}

Couzin *et al.* presented a more biologically realistic model to simulate the swarm’s collective behaviors arising from local repulsion, alignment and attractive trending on the basis of the location and direction of the individuals relative to their neighbors [29]. Couzin’s model defined three nonoverlapping behavioral zones consisting of the ‘zone of repulsion’ (zor), ‘zone of orientation’ (zoo) and ‘zone of attraction’ (zoa). Through the adjustment of parameters in simulation, the collective behaviors could possess the characteristics of natural groups. The desired state of each individual is updated as


(3a) \begin{eqnarray*} {{\mathbf {d}}_{r}}( t+\tau )=-\sum _{j\ne i}^{{{n}_{r}}}{\frac{{{\mathbf {r}}_{ij}}( t )}{| {{\mathbf {r}}_{ij}}( t ) |}},\qquad j\in \text{zor}, \end{eqnarray*}

(3b) \begin{eqnarray*} {{\mathbf {d}}_{o}}( t+\tau )=\sum _{j=1}^{{{n}_{o}}}{\frac{{{\mathbf {v}}_{j}}( t )}{| {{\mathbf {v}}_{j}}( t ) |}},\qquad j\in \text{zoo} , \end{eqnarray*}

(3c) \begin{eqnarray*} {{\mathbf {d}}_{a}}( t+\tau )=\sum _{j\ne i}^{{{n}_{a}}}{\frac{{{\mathbf {r}}_{ij}}( t )}{| {{\mathbf {r}}_{ij}}( t ) |}},\qquad j\in \text{zoa}. \end{eqnarray*}

Numerous variants of the above models considering the realistic situations have been designed. On this basis, the swarm models have been tested on the real hardware of swarm robotics [30].

**Table 1.** Mapping mechanisms from the biological swarm to the unmanned system swarm.

Characteristics	Biological swarm	Unmanned system swarm
Distribution of the organization	No central nodes Each individual interacts with neighbors	No command control center Each unmanned system makes decisions autonomously
Simplicity of the individual	Simper perception and motion abilities Simple behavior rules	Small size, low cost, partial sensors or loads equipped
Flexibility of the action mode	Adaptive to the environmental changes and able to avoid predators	Adaptive to the environment with incomplete information, uncertainties and dynamics
Intelligence on the swarm level	The swarm possesses high efficiency and emergent intelligence	Multiplied operational capabilities and improved survivability are obtained under amplification effects

## SWARM INTELLIGENCE

*Swarm intelligence* (SI) is defined as the emergence of consistent global collective motion from swarm cooperative behaviors through local interaction with the environment in [31]. The swarm consists of a large number of homogenous, unsophisticated entities without any central control or management. Each entity in the swarm executes quite simple and most often repetitive tasks. They interact with the environment and each other with the aim to conquer individual cognitive restrictions and to enable a global behavior to emerge [32,33].

The establishment of SI models is based on the use of natural mechanisms to attain comprehension of the patterns and rules resulting in flock behaviors. It has already been observed in animal systems comprising birds, fish or insects, but, though the aim of the swarm is sometimes rather complicated, each individual utilizes relatively simple rules, and interacts with their surrounding environment and individuals. Typically, one single flock individual has insufficient ability to find one superior solution, while the swarm is able to under appropriate conditions [34]. More specifically, inspiration for SI is not constrained to nature swarms, but rather to any complicated artificial system consisting of interaction entities that possess excellent characteristics, including regulation, homeostasis, collective decision-making and periodic patterns. The aim of SI is to understand the conditions, rules and interaction patterns that are able to promote the emergence of collective swarm behaviors.

Wang and Beni first introduced the term ‘swarm intelligence’ in 1989 to describe the dynamics of swarm robots that can be framed as a form of intelligent collective behavior [35]. This signifies that swarm collective behaviors began to be studied beyond the fields of nature sciences. Conversely, such comprehension can be used to cast light on how collective behaviors emerge in natural swarms and to introduce new artificial systems with swarm intelligence. There are five principles for SI algorithms to adhere to: proximity, quality, diverse response, steadiness and adaptability [36]. Firstly, the population is able to implement basic time and space computations. Secondly, the population is able to react to quality elements in the surroundings. Thirdly, the population does not need to commit its activities along exceedingly narrow channels. Fourthly, the population does not need to change the swarm behavior mode along with the variation in the environment. Lastly, the population is capable of changing the behavior mode under an appropriate computational price. Swarm intelligence, i.e. collective intelligence, has two basic properties: the amplification effect on individual intelligence and the scalability to the number of participating individuals [37]. A large swarm can significantly amplify individual intelligence. From the perspective of interaction, the essence of swarm intelligence can also contain the following three key points.

1. Exploration: swarm individuals independently explore the space for the current problem and obtain a series of information.
2. Integration: all information explored by individuals is merged in some way.
3. Feedback: the individual is stimulated to continue exploring by the feedback of merged group information.

After extensive exploration by researchers, swarm intelligence progressively evolved into two main branches: the swarm intelligence algorithm and the distributed swarm system. As Dorigo defined, swarm intelligence includes the trial design of algorithms and devices for solving distributed problems under the inspiration of collective behaviors in social animal colonies [38]. Swarm intelligence algorithms refer to a form of nature-based optimization algorithms designed to model the cooperative behavior of animals within specific communities for solving optimization problems. The most popular SI frameworks, such as the particle swarm optimization algorithm [39] and the ant colony optimization algorithm [40], consist of a swarm of minimalist agents that are modeled with the individual properties of nature societies. The spread of the concept of artificial systems began with research on swarm robotics, which is well defined by Şahin and Spears [41]: ‘Swarm robotics is the study of how a swarm of relatively simple physically embodied agents can be constructed to collectively accomplish tasks that are beyond the capabilities of a single one’ and ‘Swarm robotics emphasizes self-organization and emergence while keeping in mind the issues of scalability and robustness’. A distributed swarm system refers to the employment of swarm intelligence technologies for the analysis of swarm behaviors where the entities are physical robots, including aerial robots, ground robots, marine robots and other platforms. By taking inspiration from social animals, distributed swarm systems aim to develop robotics systems with similar swarm intelligence features that also characterize social animals [42]. Thus, a large number of robots with quite simple behaviors could achieve the desired collective behavior through local interactions with neighbor robots and the environment.

The design of a swarm-robot system with SI has faced many challenges with respect to the employment of the biological inspiration tool. From the perspective of an engineering application, it is indispensable to design the self-organization structure, local communication mechanism and feedback control method.

### Self-organization structure

The significance of a self-organization design needs to be emphasized since the system could achieve the complex task via simple rules of the individual. It is therefore highly desirable to seek self-organization behaviors in a swarm robotic system, as they can be obtained with minimal cost. Therefore, self-organization behaviors are supposed to be obtained in the swarm robotic system. Nevertheless, the definition of simple local rules for each individual is particularly challenging since it has an indirect relationship with complex global properties. To design the control system of the self-organized robotic swarm, definitions of the individual rules need to be given to promote the system’s desired pattern. The robotic swarms possess superiorities over a single robot in redundancy and the ability to handle multiple tasks simultaneously. The definition of individual self-organized behavior should adapt to the dynamic situation to promote the robustness of the swarm system. Through the self-organized rule, if a robot stops functioning, one in the swarm can replace it with no significant influence upon the system.

### Local communication mechanism

In fact, global behaviors emerge from local interactions that have not been coded directly in the individual’s behavior rules. Thus, it is necessary to discover the interaction relationship among individuals and the environment resulting in self-organization. Then, the desired global behavior should be decomposed into simple individual behaviors and their interactions with neighbors. The stigmergy mentioned above is a core concept of the biological community in nature inspired by the nesting behavior of termites, defining the information coordination mechanism of the self-organized individual [43]. Stigmergy can be regarded as an indirect or implicit communication mechanism to provide an efficient cooperation mechanism for simple individuals lacking memory and communication capabilities. Designing an implicit communication mechanism and combining it with traditional explicit communication can effectively solve the circumstances of communication conflicts and deadlocks. From the perspective of information flow, designing the local interaction mechanism can not only enhance the coordination ability and robustness of the swarm system but also improve communication efficiency to break through the bottleneck effect of communication.

### Feedback control method

From the perspective of control theory, feedback is one of the basic intrinsic elements of autonomous swarm behaviors, including both positive and negative feedback. Positive feedback strengthens the weak response of the swarm initially and urges this swarm to cope with change in the external environment. Negative feedback acts as damping to suppress the disturbance input. Positive and negative feedback respectively promote rapidity and stability. The balance between the two elements enables the group to respond quickly to the dynamic environment and remain stable in the face of uncertain disturbances.

**Figure 2.**
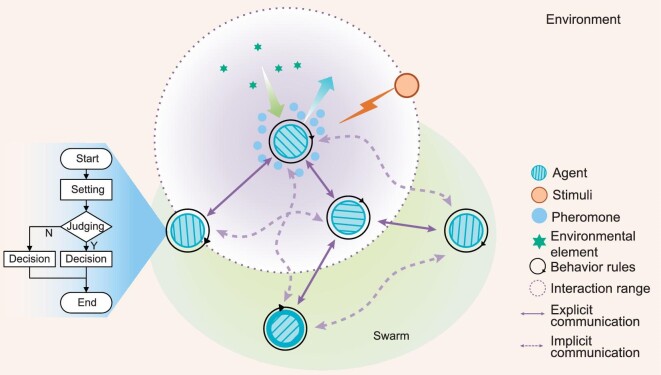
The structure of a self-organized swarm system.

The structure of the self-organized swarm system is illustrated in Fig. 2, displaying a global behavior via interaction with the environment. Aiming at the process of programming, the method is employed to decompose the process into two steps [44]. Firstly, global behavior is composed of individual behaviors and local interactions between their neighbors and the environment. Next, explicit and implicit communication modes among the individuals are defined and the mechanism of information perceiving and pheromone releasing is set between the individual and the environment. Furthermore, positive and negative feedback mechanisms are designed under the stimulus in the environment. Last, these phases are coded into the control program embedded into each individual. This process is complex because the definition of individual behavior rules should correspond to the desired swarm model. Thus, it is common to take inspiration from the biological community to simplify decomposition from global cooperative behaviors to individual interaction rules. In the case of unmanned aerial vehicle (UAV) close formation inspired by the ibis V-formation migration, four steps are consistent with the above structure. Firstly, the UAV motion of the close formation is decomposed into the leading-following relationship of the pairwise individuals. Secondly, the direct reciprocation model is constructed for individual interactions. Thirdly, the fuel cost or the flight length conditions are defined to trigger the pairwise position switches in the UAV swarm. Lastly, the above three steps are programmed in the same way for each individual.

## UNMANNED SWARM SYSTEMS

### Aerial robotics swarm

A swarm of UAVs is a group of aerial robots working cooperatively to accomplish the given aim [45]. Each UAV can be controlled by ground stations and remote control units in hand, or processors loaded on the aircraft autonomously. On this basis, the UAV swarm can be classified into fully autonomous and partly (semi-)autonomous swarms. From another perspective, the classification can be divided into centralized, distributed and hybrid control swarms. In the centralized control swarm, the drones are positioned in the hierarchy of multiple layers. Each drone receives commands from its leader individual at the upper layer rather than making its own decisions. The ground station plays the role of the top layer in the hierarchy. For this structure, it is easy to implement the task algorithms, but it possesses problems such as poor real-time and anti-disturbance performance. In the distributed control swarm, all drones are positioned in a single layer and regarded as their own leader with independent decision-making capability. This structure could overcome the boundedness of the centralized structure, which is robust and adaptive to the dynamic complex environment. In hybrid swarms that possess both centralized and distributed characteristics, drone nodes are grouped and assigned by function, type, authority, etc. The nodes located separately in the higher and lower layers form the centralized control swarms and the nodes in the same layers form a distributed control swarm. Hybrid control swarms are established to be clear on the division of labor, highly stable under disturbance and easy to maintain, but their structure is complicated to design.

In Unmanned Aircraft Systems Roadmap 2005–2030 issued by the Office of the Secretary of Defense, an onion-like layered series of capabilities has been adopted to define ten levels of autonomy. The definitions handle remote operation and preprogrammed flight by a single aircraft to autonomous swarm flight [46]. Small Unmanned Aircraft Systems (SUAS) Flight Plan 2016–2036 issued by the United States Air Force emphasized the broad prospects and high value of small unmanned aerial systems from the strategic level. And new concepts of using SUAS to realize tactical- to strategic-level mission goals are augmented or redefined, including swarming, teaming and loyal wingman, corresponding to machine-to-machine, man-to-man, man-to-machine means. China Electronics Standardization Institute successively promulgated the white paper on the development of intelligent unmanned swarm systems in 2021 and group standard of information technology unmanned swarm terminology in 2022, which put forward the urgent need to establish a unified intelligent unmanned swarm technology system and standard system for the guidance of industry development in the product life cycle of design, development, operation and maintenance [47,48]. These all send a signal that the development trend of UAVs is from a single drone to multiple drones and UAV swarms, and that the control architectures of UAV swarms step from centralization forward distribution. Through efficient cooperation, autonomous UAV swarm systems can embody better coordination, intelligence and autonomy than manual systems. The cooperation of a UAV swarm has the following characteristics, leading to discernible advantages in situation awareness, task efficiency, etc.: (1) solves the conflicts between multiple UAVs in the same space effectively; (2) possesses efficient information sharing, fault resistance and self-healing capabilities via the decentralized communication network; (3) obtains high decision accuracy with distributed swarm intelligence; (4) improves the detection accuracy of active and passive detection by adopting the distributed detection method. For the advancement of cooperation technology, there are four main aspects that need to be executed.

#### Aerial launch and recovery

To satisfy the needs of long-range, low-cost, multi-wavelength, hierarchical mission execution capabilities of large-scale UAV swarms, aerial launch and recovery technology have rapidly developed. The ‘launch-work-recovery-relaunch’ mission execution mode can significantly improve the efficiency ratio and the sortie efficiency of autonomous UAV swarms. Aerial launch usually considers two methods: ejection and distribution. The current aerial launch projects are mostly conducted with a large transport plane or bomber that flies to the predefined location carrying a large number of small UAVs, drops them to perform reconnaissance, attack or interference tasks, and then recovers them after the tasks are completed or battery power is low. In the tests carried out on October 25, 2016, three US Navy F/A-18 Hornet two-seat variants successfully released a ‘swarm’ of 103 Perdix semi-autonomous drones during flight. On March 19, 2019, NASA tested a swarm of 100 US Navy Cicada drones released by four large drones (called Hives). The Cicadas are mounted on the underside of a Hive drone and then released on demand with a mechanical switch [49]. As announced in September 2020 by China Academic of Electronics and Information Technology, the land launch and air launch of a fixed-wing UAV swarm have been verified, reflecting the capabilities of formation reconfiguration, ground observation and attack, precision strike and others. The U.S. Defense Advanced Research Projects Agency (DARPA) Gremlins program has been exploring the concept of employing large aircraft as the launch platforms for recoverable UAV swarms since 2016. On October 29, 2021, a Lockheed C-130A Hercules aircraft launched and recovered an X-61A Gremlins air vehicle in flight for the first time in Utah. C-130 could employ two recovery approaches to catch an X-61 Gremlins drone in flight: a mechanical arm or metal cable [50].

#### Communication network

Facing the challenges of complex battlefield environments, such as land, sea, air, space, electromagnetic and network, establishing reliable communication networks among the UAV swarm is the key to carrying out cooperative tasks. Generally, the reliability of the communication network has many challenging parameters, such as time delay [51], switching topologies [52], node-link interruption [53], presence of jammers [54] and unpredictable noisy channels [55]. In the aspect of theoretical exploration, the communication network of a UAV swarm is usually considered via the graph theory method, which introduces the network, node and link as a graph, vertex and edge, respectively. At the same time, flight tests in the real environment have also made phased progress. As demonstrated on August 27, 2015 at Camp Roberts, Timothy Chung’s group at the Naval Postgraduate School achieved an autonomous swarm of 50 UAVs, among which each individual is equipped with three communication systems to establish the network system [56]. The swarm of CH-4 UAVs successfully completed a number of flight missions in 2016 through sensing and cooperating, such as over-the-horizon flight, line-of-sight relay of satellite communication and simultaneous transmission of multi-channel satellite communication. In 2017, 119 small fixed-wing UAVs completed the autonomous formation tasks released by China Academic of Electronics and Information Technology with the hierarchical clustering approach to build a self-organized network.

#### Decision-making and control

Based on the interaction topology network, the UAV swarm could carry out information aggregation and fusion, make decisions on the next mission and move on to complete the current action [57]. First, the UAV individual could acquire situation elements at the current time and space environment, fuse and analyze the information, and obtain the predictive inference of the states at the next moment to establish situation awareness. However, the actual battlefield environment faces challenges, such as incomplete information, the existence of interference, deception and attack signals [58]. Second, the decision-making process based on the environment cognition arranges the UAV swarm to execute the mission at a specific pattern depending on the dynamic task allocation and coordinated control mechanism [59]. Correspondingly, the task assignment algorithm and interaction mode are supposed to be well designed to support the swarm cooperative decision-making process, which is recently inspired by biological swarm behaviors in various applications [60]. Third, UAV swarms take action to perform the assigned tasks via different configurations of formation control. There are several key technologies necessary for autonomous flight, including formation maintenance [61,62], formation reconfiguration [63,64], obstacle avoidance [65] and energy conservation [66].

#### Technical application and verification

The maturity of cooperative algorithms and technologies requires multiple iterations of technical application and validation experiments. The research group in Hungary proposed a flocking model that ensured seamless navigation in confined spaces, verified with 30 real drones in 2018 [67]. Researchers in China developed miniature but fully autonomous drones and palm-sized swarm platforms with onboard perception, localization and control capabilities by imitating birds for trajectory planning approaches in 2022 [68]. The China Academy of Electronics and Information Technology reportedly carried out the experiment with 200 fixed-wing drones simultaneously launched to switch between various configurations and carry out reconnaissance and attacking missions on ground targets in 2018 [69]. The US Army conducted a 2022 Experimental Demonstration Gateway Exercise (EDGE 2022) from late April to early May to test interoperability, the network, electronic warfare, multi-intelligence sensors, interactive drone swarming and enhanced sustainment on the largest interactive drone swarm to date, redefined as ‘Wolfpack’ [70].

### Ground robotics swarm

Ground robots in a swarm have always intrigued researchers and engineers due to their wide application in military operations, social activities, intelligent manufacturing, etc. Besides the advantages shared by aerial robots, ground robots play a key role in restricted space and environments with portability, concealment and flexibility. There are three frontier research topics in three different operation spaces.

#### Micro-nano robot swarm

Micro- and nanorobotics is an emerging field of the research arising from the cross-fusion of micro-/nanotechnologies and robotics [3]. Compared with macroscopic robots, micro- and nanorobots could conduct tasks on an exceedingly small scale because of the characteristics of low weight, small size, large thrust-to-weight rate, high sensitivity and high flexibility. Thus, micro- and nanorobots have intrigued researchers and opened up numerous application frontiers, including drug delivery, disease diagnosis and minimally invasive surgery. As a single micro-nanorobot is limited in size and function, a micro-nanorobot swarm has gradually become the research focus, promoting the trend of cooperation. Swimming micro-robots that gain energy supply from external magnetic fields show various intelligent collective behaviors, varying from self-organized to cooperative movement. A strategy for the reconfigurable magnetic micro-robot swarm is presented to emulate the cooperative mechanisms and self-organization phenomena of natural swarms. The strategy utilizes alternating magnetic domains to program the hematite colloidal particles into ribbonlike, chain, liquid and vortex micro-robotic systems and enables speedy and reversible changes between them [71]. Moreover, present micro-robot swarms lack intelligent behaviors to autonomously regulate their distribution and adapt to environmental change. An autonomous environment, accommodative micro-robot swarm is designed using the deep learning-based real-time distribution planning method [72]. Each robot possesses the real-time appropriate decision-making capability for unknown and unstructured environments. For environment-adaptive micro-robot swarm navigation, four different autonomy levels are defined and the corresponding system components are designed.

#### Manipulator swarm

The cooperation among multiple industrial robots will bring cooperative motion planning and synchronous motion control problems of multiple manipulators under constraints [73]. In multi-manipulator cooperative motion planning, it is essential to customize the cooperative manner, in order to safely and efficiently achieve the desired manipulation task. The manipulator in the space station is one of the key pieces of equipment for the construction, operation, maintenance and expansion of the space station. With the growing complexity of space tasks and the development of related technologies, space manipulator technology presents a trend of configuration from single-arm operation to multi-arm operation. The Japanese Experiment Model Remote Manipulator System (JEMRMS) consists of two arms: the main arm (MA) and the small fine arm (SFA) [74]. The end effector of the MA can grapple the common grapple fixtures in the space station and the SFA can perform more dexterous tasks than the MA. The China Space Station manipulator consists of an arm in the core module and another arm in the laboratory module [75]. The two arms can work independently or cooperatively or can be combined as one arm to expand the operating range. The synchronous motion control problem is another research focus. The problem of distributed control of multiple redundant mobile manipulators is tackled by a distributed proximal gradient algorithm [76]. The formation control tasks are introduced as equality constraints with the variables being the velocities. Then the manipulator swarm could collectively transport an object tracking a desired trajectory with energy and manipulability optimized. The cooperative problem of the multi-manipulator could be considered as a multi-agent system. The key problem to solving the consistency and coordination control is to design appropriate protocols or algorithms to ensure consistency of each manipulator and the stability of the system [77]. In the future, space manipulators with multi-manipulator configurations will be developed to complete more complex operations through multi-manipulator cooperative movement.

#### Unmanned ground vehicle swarm

The cooperation of unmanned ground vehicles (UGVs) in a swarm puts forward requirements of precise positioning and navigation technology in both indoor and outdoor environments. The outdoor environment supports positioning and navigation devices, such as GPS, IMU, electronic compass and other sensors. However, the lack of prior information and GPS signal greatly increase the difficulty of navigation and positioning technology in indoor or GPS-denied environments. Thus, it is suitable to utilize vision-based systems as an alternative for indoor environments [78]. Therein, visual Simultaneous Localization and Mapping technologies [79] have the characteristics of small size, light weight, high precision, better real-time performance, low cost, etc. Each UGV in the swarm interacts with other individuals to share environment information and optimize the task assignment, so as to improve the overall operational effectiveness via the cooperative mechanism. Therefore, it is necessary to conduct a deep study about autonomous path planning, obstacle avoidance, cooperative consensus control and other technologies [80]. The exploration and verification of the swarm cooperative algorithm are developed more in-depth with the UGV system. Swarm intelligence is also widely regarded as the key technology to improve the capacity of swarm systems. E-puck robots, which were unveiled by École Polytechnique Fédérale de Lausanne, have been employed to test the controllers on the capability to handle the noise in global positioning data and the robustness of the swarm with the form of broadcast messages [81]. Kilobot robots designed by Harvard University realized programmable self-assembly of complicated two-dimensional shapes in a thousand-scale swarm through local interactions and cooperative algorithms [82]. Particle robotics are controlled to achieve autonomous locomotion, phototaxis and object transport via the simple distributed algorithm inspired by collective cell migration phenomena in biology [83]. With the appearance of intelligent UGV like BigDog [84], various swarm robotics competitions like the DARPA Subterranean Challenge and Swarmathon have risen to greatly propel the development of multiple UGV systems [85,86]. The swarm of cooperative UGVs, as extensions of human hands, eyes and ears, could assist them to conduct more complicated tasks, particularly for hazardous tasks like bomb disposal. DARPA’s OFFensive Swarm-Enabled Tactics (OFFSET) program deployed swarms of autonomous air and ground vehicles to demonstrate a raid in an urban area during the agency’s third field experiment in 2022 [87]. Shenyang Institute of Automation, Chinese Academy of Sciences demonstrated a new mode for onsite security of large-scale mass events based on the cooperation of unmanned aerial and ground systems in 2021.

### Marine robotics swarm

Unmanned marine vehicles (UMVs) refer to marine robots moving above and below the water with no operation from the human on board. UMVs are usually equipped with the necessary sensors and payloads to accomplish neither civilian or military missions, such as environmental monitoring, target surveillance and region reconnaissance.

A UMV consists of unmanned surface vehicles (USVs) and unmanned underwater vehicles (UUVs). USVs are a type of unmanned surface vessel that can be fully autonomous, semi-autonomous or switched to manual control. They are able to navigate autonomously and avoid obstacles intelligently by following the current parameters and waypoints obtained from programs. This makes them ideal for data collection, autonomous hydrographic surveys and military applications. UUVs are supposed to carry out tasks without real-time supervision and intervention from the operator. They are capable of cruising on a predetermined course, publishing and receiving the information and making decisions according to the posture change driven by the embedded program. UUVs mainly incorporate remotely operated vehicles (ROVs) and autonomous underwater vehicles (AUVs). The development of ROVs could overcome the limitations of the operators and manned marine vehicles. Therefore, they are remotely operated by crew on nearby vehicles and connected to their operating bases through an umbilical cord link. This link can provide lines and power supply, communication and data links. ROVs are usually placed in four categories according to their size: work-class ROVs, observation- or inspection-class ROVs, mini- or micro-ROVs. They can be employed in military and civilian scenarios, such as pipeline and offshore platform inspection tasks. AUVs can perform a task lasting several months without direct real-time control. They can execute the preprogrammed, predefined waypoints normally and make decisions on the emergent circumstances via intelligent algorithms. Most AUVs are powered by battery and propelled by thrusters. Therein, the underwater gliders use buoyancy changes to adjust the depth and work on sampling or monitoring tasks. AUVs with no link to the ground possess high operability and can travel to remote positions and through narrow complicated pathways [88]. Other AUV applications include oil and gas exploration, seabed surveying and anti-submarine warfare.

Compared with land and air environments, the ocean water environment has more disturbances, such as ocean currents, faults and complex submarine topography. Underwater acoustic communication is an effective form of underwater communication, but the propagation speed of the acoustic wave is 5 orders of magnitude lower than that of electromagnetic waves, leading to problems such as low information transmission speed, serious delay, packet loss and fast attenuation. Meanwhile, it is difficult for one single UMV to conduct complicated tasks. However, the AUV swarm could implement tasks cooperatively to achieve greater efficiency in the complex marine environment [89]. Thus, multi-UUV cooperation will improve the intelligence and efficiency of multiple individuals performing tasks independently, enabling them to better accomplish tasks that cannot be conducted by one individual. There are two key technologies in the cooperation of a UUV swarm.

#### Communication technology

Connected communication is the basis of reasonable and efficient cooperation between UMVs. Because the underwater environment has a strong attenuation effect on electromagnetic waves and light waves, long-distance communication relies heavily on underwater acoustic communication at present. Underwater acoustic communication is realized by the underwater acoustic communication machine carried by UMVs, and the communication content can be request, response, task, target, state information, control instruction and so on. However, the acoustic wave will generate a large transmission delay, so real-time communication between UMVs is difficult to achieve. Moreover, the transmission distance of acoustic waves is limited by the carrier frequency and transmitting power. The scattering, transmission loss and echo interference of acoustic waves in water have effects on the distance and quality of underwater acoustic communication. Thus, experts in related fields have done a lot of research to explore communication methods, such as IR sensors [90], blue light [91] and computer vision [92].

#### Navigation and location technology

Navigation and location technology is the prerequisite and key technology of cooperation in the multi-UMV swarm. However, due to the complex underwater environment and the limitations of UMV itself in size, the methods to achieve multi-UMV locations are limited. They mainly include the following.

1. Acoustic navigation location: although this method has high accuracy, the cost of the baseline layout is high and the location accuracy is limited by the coverage of the baseline.
2. UMV navigation presumption: this method is characterized by simple operation and low cost, but the location error accumulates over time.
3. Inertial navigation: this method possesses high precision with the expensive cost of high precision inertial equipment, especially in a large-scale group.
4. Geophysical guidance (gravity matching, geomagnetic matching, etc.): this method needs to provide a corresponding matching database, which is not suitable for unknown regions.
5. Multiple-UMV cooperative location: a small number of UMVs are equipped with high-precision navigation equipment to provide accurate relative positioning information for other UMVs. Other UMVs utilize relative positioning information to correct their location errors. Multiple-UMV cooperative navigation has the characteristics of moderate cost and simple implementation, which can meet the needs of the rapid location. However, cooperative navigation usually relies on underwater acoustic communication for location information transmission, resulting in a large transmission delay.

Many scholars have made significant contributions to theory research and hardware implementation of multi-UMV cooperation. For cooperation control, multiple UMVs are supposed to meet task requirements and environmental constraints. Because of environmental disturbance, discussions on this topic have been conducted in view of weak communication topology [93], communication delays [94], ocean current disturbances [95], navigation and localization [96], etc. Under the physical limitation of UMVs, circumstances such as uncertain dynamics, parametric uncertainties and actuator saturation are inevitably to be considered [97–99]. As for the complex coordination of centralized explicit communication, implicit or hybrid centralized coordination approaches have arisen, inspired by the collective behaviors of fish schooling [100,101]. At the practical level, there are several significant efforts to deploy underwater swarm cooperation. Nekton Research Institute in the UK is continuing with the advancement of micro-UUVs and the underwater multi-agent platform. The platform comprises N-UUVs and a supporting infrastructure that has been employed in the research of distributed search, formation control, oceanographic survey and other related research [102]. DARPA’s Collaborative Networked Autonomous Vehicles program has been deployed from 2009 and utilizes a shared acoustic network to distribute data for underwater target detection, location and tracking missions. The system, coupled with a stationary passive sonar node, is used in the distributed agile submarine hunting system to detect submarine targets over a wide area [103]. Exercise Unmanned Warrior organized by the Royal Navy and its partners in academia and industry is a display of autonomous robotic systems that carry out a dazzling array of air, surface and sub-surface tasking, from underwater surveying to mine countermeasures. China Ship Scientific Research Center has conducted three sea trials for formation networking tasks of ‘Haixiang’ underwater gliders. The tests obtained temperature, salinity, oxygen concentration, chlorophyll and other marine environment measurement data in relevant waters, covering an area of 112 square kilometers.

## HUMAN-MACHINE SYSTEM

**Figure 3.**
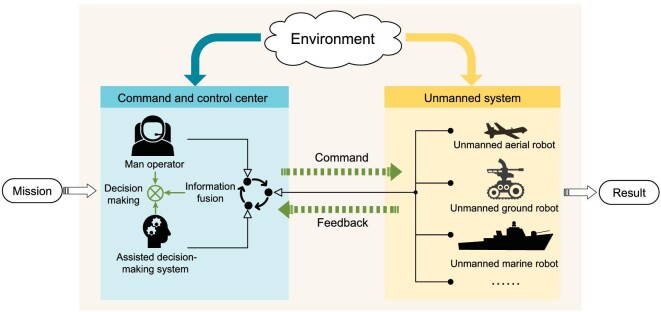
Tri-Co framework of the human-machine-environment system.

In biology, the experiments of bird flocks under human intervention provide an innovative way to further study the mechanisms of flock movement and communication interaction. The free flight experiments for long distances have accomplished human control of the robo-pigeon by neural stimulation [104]. Then, contrast experiments have been conducted on the basis of brain micro-stimulation technology to design the controlled variables. The robo-pigeon with the higher hierarchical level may effectively balance their preferred directional choice in the flock [105]. Nowadays, biological experiments where a robotic falcon dashes into the pigeon flock help researchers to analyze the species-specific pattern of collective escape behavior [106]. Because of the possibility of introducing human factors into a natural bird flock, it is more hopeful for us to further understand the collective behavior and mechanism from the animal behavior paradigms. Because of the capability limitation of the unmanned system in the aspects of situational awareness and real-time decision-making, the cooperation of human wisdom and unmanned systems qualifies as a force multiplier and improves the level of autonomy and intelligence to meet the growing challenges in complex environments. Specifically, combining the advantages of manned and unmanned systems could strength the task efficiency and effectiveness, while offering security and lower risk to operators and assets simultaneously. Figure 3 shows the coexisting-cooperative-cognitive (Tri-Co) framework incorporating the manned operating platform and unmanned systems into one team. The mission aim of the system is defined by a command-and-control entity (C2) regarded as information input [107]. The environment perceived by platform sensors is considered as input of the command and control center and unmanned systems to provide situational information. There exist different roles in the team. The human operator has the highest authority and accomplishes decision-making tasks with suggestions from the assisted decision-making system (ADS), i.e. an automated planning aid [108]. Unmanned systems are delegated to accomplish the tasks by the commands directly from human operators or the ADS. This Tri-Co framework helps operators to supervise and control unmanned systems with a maintainable workload.

U.S. Army Unmanned Aircraft Systems Roadmap 2010–2035 issued in 2010 covered a 25-year period, divided into three distinct periods: near term, mid term and far term. The far term emphasized drastic commonality and capability improvements of both manned and unmanned systems to lay the foundation for man-machine cooperation. SUAS Flight Plan 2016–2036 proposed the concept of a loyal wingman, highlighting the man-to-machine means. Unmanned Systems Integrated Roadmap 2017–2042 addressed the critical need of interoperability to promote the capability synergy of manned and unmanned systems in 2018. The artificial intelligence strategic development plan announced by the Chinese State Council in 2017 covered man-machine cooperation eight times and considered it one of the key technologies for establishing the key generic technology system in the area of artificial intelligence. Professor Tianran Wang pointed out that the next generation of robots will cooperate with the human in the Opening Ceremony of the 2018 National Robot Development Forum and RoboCup China competition. In the 2018 IEEE/CSAA Guidance, Navigation and Control Conference, Professor Bangkui Fan pointed out that the high degree of cooperation between manned and unmanned systems is the key to realizing the practical application of a UAV swarm. Besides, the National Natural Science Foundation of China launched the first major research program in the field of robotics in China on the advice of experts, including Han Ding, Xuejun Yang and Nanning Zheng, to promote basic research on Tri-Co robots. Thus, it has become an important development trend to adopt the cooperation of manned and unmanned systems to realize the multiplication and even exponential increase of the task capability in cross-domain platform systems.

Aiming at achieving the autonomous cooperation of the manned-unmanned system, Professor Jie Chen proposed research challenges containing four levels [109].

1. System level: organizational structure and collaboration mode.
2. Decision-making level: task allocation and behavior planning.
3. Control level: cooperative motion control.
4. Security level: security of command control.

Zheng *et al.* proposed a vision of human-centric networked unmanned systems: the unmanned intelligent cluster. Cooperation can be achieved by knowledge sharing and social awareness between distributed unmanned systems and humans [110]. Generally, the presence of a human operator could contribute to (1) recognizing and mitigating the boundedness of autonomous unmanned systems; (2) obtaining the critical information unavailable to unmanned systems to improve mission effectiveness; (3) conveying the changes of task goals timely [111]. The control methods that an operator exerts on the swarm can be summarized as (1) the operator could control the unmanned system via an algorithm and behavior selection, while the unmanned swarm has a high degree of autonomy [112]; (2) the parameter setting method appears in most cases with indirect effects on the swarm [113]; (3) the operators influence the swarm via the direct or virtual environmental factors [114]; (4) the operators select to control a subset of leading individuals to reduce control complexity [115].

The level of swarm autonomy (LOA) can be roughly divided into three ranks: manual, mixed-initiative and fully autonomous LOA [116]. Three basic modes of unmanned-manned cooperation under the confrontational situation are given in [117]. A fully centralized mode represents the unmanned systems cooperating with the human operator. The finitely centralized distribution mode represents that the unmanned systems are assisted by the human operator. The acentric distribution mode represents that the unmanned systems and human operator complement their skills. Five different levels of interoperability are defined more explicitly by NATO Standardization Agreement 4586 (STANAG 4586) to describe how operators control both the UAVs and the payload.

- Level 1: indirect transmission/receipt of UAV-related metadata and data.
- Level 2: direct transmission/receipt of UAV-related metadata and data.
- Level 3: control and monitoring of UAV payload, rather than the unit.
- Level 4: control and monitoring of the UAV, with no launch and recovery.
- Level 5: control and monitoring of the UAV, containing launch and recovery.

It can be seen that level 5 requires the installation of a fully remote pilot station in the manned aircraft and offers the crew maximum control.

In the manned and unmanned air systems, providing pilots or operators the ability to control UAS enables them to take full advantage of ISR (intelligence, surveillance, and reconnaissance) capabilities to enhance decision-making and improve safety during dull, dirty and dangerous tasks. As for manned and unmanned marine systems, divers may cooperate with UMVs to complement each other to accomplish the tasks of underwater salvage, rescue, maintenance and scientific research together. A variety of devices equipped by the UMVs could reduce divers’ loads, and enhance the flexibility and automation level, such as communication modules, multi-beam and side-scan sonar, and the ultrashort baseline location system. Meanwhile, UMVs are supposed to assess the physical condition of the divers via the connection with life detection and support systems. Thus, divers have the opportunity to escape dangers or accidents under the alarm and assistance of UMVs. The design of the human-UMV cooperation system should take the role of operators in complex tasks into account to be flexible and applicable, and, more importantly, adopt compliant control methods to guarantee the safety of divers while UMVs approach divers. To solve the complicated and uncertain situations with the human-UMV cooperation system, one of the foremost considerations must be the group role assignment problem. Through role-based collaboration methods, researchers could establish an efficient and flexible system [118]. In manned and unmanned ground systems, automation is achieved via the heterogenous system of UGV platforms and human decision-making to conduct the work with one excavator operator and one construction planner. The use of UGVs plays a special role in the reduction of human exposure in dangerous zones. The wide application of UGVs raises the requirement for mobile platforms to meet certain quality requirements and to also be operated easily by human operators. In the TianGong-2 (TG-2) spacelab mission, a human-robot collaborative on-orbit servicing experiment was conducted as one of the three key tasks. The TG-2 robotic task is designed to complete various prototypical experiments under a micro-environment to validate key technologies of space robots and on-orbit human-robot collaboration, which gain experience and experimental data about robotic on-orbit servicing by assisting or cooperating with human astronauts [119]. Thus, human-machine interfaces could be enhanced to allow easier machine operation and interaction. Then, the autonomy level, including the ability of sensing, perceiving, communicating, analyzing, decision-making and action, could be increased.

## FUTURE PROSPECTS

**Figure 4.**
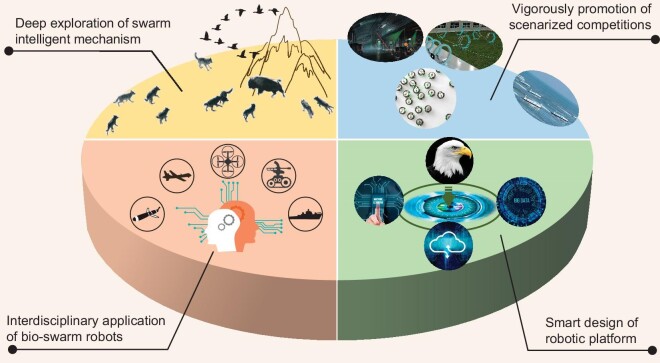
Four development directions of the swarm-robot system.

The development directions of the swarm-robot system are preliminarily discussed in this section, as shown in Fig. 4.

### Deep exploration of the swarm intelligent mechanism

Research on the mechanisms of large-scale swarm movement of biological groups, such as birds and fish, has attracted extensive attention from researchers in different fields. Through simple rules and local interactions, biological groups show collective behaviors with strong robustness, high self-adaptiveness and good scalability, which are the desired characteristics of a swarm-robot system. Although we have a primary understanding of the collective mechanisms in biological swarms through theoretical modeling and empirical analysis, the self-organizing emergence mechanism of swarm intelligence still requires further research. Currently, swarm-robot systems pay more attention to the scale effect, hoping to suppress the enemy with the number advantage through cooperation. However, this line of research is only similar to animal communities in form and intuitionistic. Employing the intelligent mechanisms emerging from the biological swarm behaviors can improve the system efficiency to a greater extent and achieve exponential growth of task effectiveness. In this sense, research on the theory and method of biological swarm intelligence is becoming more and more important related to the heterogeneous group, individual learning, etc. Thus, data analysis from biological swarm experiments will help to reveal the internal mechanism of self-organizing intelligent swarm behaviors. The biological experiments under human intervention could be helpful for the exploration of the intelligent mechanisms in animal swarms. Also, the mapping mechanisms from animal collective behaviors to swarm robotic cooperation are supposed to be modeled with the task requirements.

### Interdisciplinary application of bio-swarm robots

To adapt to the requirements of platform performance, battlefield environment and tactical tasks, the incentive and convergence mechanisms of swarm intelligence in nature are explored to change the bottleneck situation of the intelligent unmanned system with intelligence but no wisdom, sensing but no cognition, specialty but no generality, cooperation but no convergence. On the one hand, the coordinated mechanisms’ inner biological swarms are supposed to be utilized in the processes of flight management and control, collaborative decision-making and information sharing. On the other hand, swarm-robot systems are supposed to be endowed with the ability of self-learning and evolution by biological swarm intelligence to gain full autonomy. The autonomous control technology of swarm-robot systems based on bionics is expected to be broken through via the cutting-edge technology research of biology, control, artificial intelligence, robotics and other interdisciplinary fields. Thus, exploring the phenomenon of swarm intelligence incentive and convergence in the natural community and applying it to the subversive technology of autonomous swarm control of robots are very compatible both in terms of the theoretical framework and application requirements.

### Smart design of the robotic platform

In the complex multi-task environment, the intelligent swarm-robot system is required to comprehensively perceive and understand the environment. At the same time, information sharing and interaction within the swarm for individual decision-making are the basis for a swarm-robot system to achieve high-level autonomous control. The task of environment sensing is required to collect information data on the robot using the load equipment, such as photoelectric and radar, discover the rules and mine the targets from the data, and then identify the targets to improve the global recognition of the complex environment situation and enhance the reliability of the system. The eagle has the sharpest visual acuity among all animals, whose powerful vision perception mechanisms bring abundant inspiration for traditional visual applications. Biological eagle eye vision technology provides a creative way to solve visual perception issues [120]. Therein, the theoretical method and application technology of bionic visual perception could promote the development of environment perception and recognition technology.

### Vigorous promotion of scenarized competitions

Drawing on experiences from the technology development model of military powers, the scenarized competition of the swarm-robot system needs to be vigorously promoted [121]. Firstly, it is important to strengthen the strategic planning of robotic swarm development from the macro-level, and integrate it into the equipment system for overall planning. There are a number of international large-scale competitions dedicated to the cultivation and exploration of civil scientific research. The more well-known unmanned aerial vehicle events include the International Micro Air Vehicles, the International Aerial Robotics Competition, the Aviation Competition and the Tri-Co Robot Challenge of the World Robot Contest. It is effective to refer to this model to explore the future intelligent swarm combat concept. Through the promotion of the scenarized competition, the swarm-robot technology has broad prospects not only in penetration reconnaissance, decoy interference, monitoring and fighting, coordinated attack and other national defense fields, but also in intelligent transportation, geological survey, disaster monitoring, agricultural protection, logistics transportation and other national economic development.

## CONCLUSION

The coordinated and ordered movements emerging from the aggregation of a large number of individuals in nature provide plenty of examples and abundant inspirations for swarm intelligence research. The kinetic model and behavior mechanism of swarm intelligence promote the development of cooperative control theory and methods of unmanned swarm systems. This article starts with an introduction of natural collective behaviors in schools, packs and flocks. Then, the connotation and mechanism of swarm intelligence are given on the basis of biological behaviors. Furthermore, the SI concept is applied in the cooperative control of three types of unmanned swarms, i.e. an aerial robotics swarm, a ground robotics swarm and a marine robotics swarm. Moreover, the intervention of human operators has led to multiple and even exponential growth of the mission capability of cross-domain platform systems. The advancement trending of swarm intelligence and the swarm system is discussed preliminarily as reference directions for subsequent exploration.

## References

[1] Qiao H , Chen JH, Huang X. A survey of brain-inspired intelligent robots: integration of vision, decision, motion control, and musculoskeletal systems. IEEE Trans Cybern 2022; 52: 11267–80. 10.1109/TCYB.2021.3071312 33909584

[2] Liang DK , Sun N, Wu YM et al. Energy-based motion control for pneumatic artificial muscle actuated robots with experiments. IEEE Trans Ind Electron 2022; 69: 7295–306. 10.1109/TIE.2021.3095788

[3] Yang J , Zhang C, Wang XD et al. Development of micro- and nanorobotics: a review. Sci China Tech Sci 2019; 62: 1–20. 10.1007/s11431-018-9339-8

[4] Arnold R , Carey K, Abruzzo B et al. What is a robot swarm: a definition for swarming robotics. In: 2020 IEEE 10th Annual Ubiquitous Computing, Electronics & Mobile Communication Conference (UEMCON), New York. New York: IEEE Press, 2020, 74–81.

[5] Croon GCHE , Dupeyroux JJG, Fuller SB et al. Insect-inspired AI for autonomous robots. Sci Robot 2022; 7: eabl6334. 10.1126/scirobotics.abl6334 35704608

[6] Schranz M , Caro GAD, Schmickl T et al. Swarm intelligence and cyber-physical systems: concepts, challenges and future trends. Swarm Evol Comput 2020; 60: 100762. 10.1016/j.swevo.2020.100762

[7] Dorigo M , Theraulaz G, Trianni V. Swarm robotics: past, present, and future. Proc IEEE 2021; 109: 1152–65. 10.1109/JPROC.2021.3072740

[8] Zhang Y , Yang Q. An overview of multi-task learning. Natl Sci Rev 2018; 5: 30–43. 10.1093/nsr/nwx105

[9] Brambilla M , Ferrante E, Birattari M et al. Swarm robotics: a review from the swarm engineering perspective. Swarm Intell 2013; 7: 1–41. 10.1007/s11721-012-0075-2

[10] Zhu YF , Tang XM. Overview of swarm intelligence. In: 2010 International Conference on Computer Application and System Modeling (ICCASM), Taiyuan, China. New York: IEEE Press, 2010, 400–3.

[11] Duan HB , Qiu HX. Unmanned Aerial Vehicle Swarm Autonomous Control Based on Swarm Intelligence. Beijing: Science Press, 2018.

[12] Yu ZQ , Zhang YM, Jiang B et al. A review on fault-tolerant cooperative control of multiple unmanned aerial vehicles. Chinese J Aeronaut 2020; 35: 1–18. 10.1016/j.cja.2021.04.022

[13] Attanasi A , Cavagna A, Castello LD et al. Finite-size scaling as a way to probe near-criticality in natural swarms. Phys Rev Lett 2014; 113: 238102. 10.1103/PhysRevLett.113.238102 25526161

[14] Nagy M , Ákos Z, Biro D et al. Hierarchical group dynamics in pigeon flocks. Nature 2010; 464: 890–3. 10.1038/nature08891 20376149

[15] Cavagna A , Cimarelli A, Giardina I et al. Scale-free correlations in starling flocks. Proc Natl Acad Sci USA 2010; 107: 11865–70. 10.1073/pnas.1005766107 20547832 PMC2900681

[16] Bialek W , Cavagna A, Giardina I et al. Social interactions dominate speed control in poising natural flocks near criticality. Proc Natl Acad Sci USA 2014; 111: 7212–7. 10.1073/pnas.1324045111 24785504 PMC4034227

[17] Voelkl B , Portugal SJ, Unsöld M et al. Matching times of leading and following suggest cooperation through direct reciprocity during V-formation flight in ibis. Proc Natl Acad Sci USA 2015; 112: 2115–20. 10.1073/pnas.1413589112 25646487 PMC4343164

[18] Portugal SJ , Hubel TY, Fritz J et al. Upwash exploitation and downwash avoidance by flap phasing in ibis formation flight. Nature 2014; 505: 399–402. 10.1038/nature12939 24429637

[19] Katz Y , Tunstrøm K, Ioannou CC et al. Inferring the structure and dynamics of interactions in schooling fish. Proc Natl Acad Sci USA 2011; 108: 18720–5. 10.1073/pnas.1107583108 21795604 PMC3219116

[20] Kimbell HS , Morrell LJ. ‘Selfish herds’ of guppies follow complex movement rules, but not when information is limited. Proc Royal Soc B 2015; 282: 20151558. 10.1098/rspb.2015.1558 PMC4614772 26400742

[21] Jiang L , Giuggioli L, Perna A et al. Identifying influential neighbors in animal flocking. PLoS Comput Biol 2017; 13: e1005822. 10.1371/journal.pcbi.1005822 29161269 PMC5697824

[22] Herbert-Read JE , Perna A, Mann RP et al. Inferring the rules of interaction of shoaling fish. Proc Natl Acad Sci USA 2011; 108: 18726–31. 10.1073/pnas.1109355108 22065759 PMC3219133

[23] Juliane B , Katharina S, Federica A. Dogs (Canis familiaris) and wolves (Canis lupus) coordinate with conspecifics in a social dilemma. J Comp Psychol 2019; 134: 211–21. 31855034 10.1037/com0000208

[24] Bonanni R , Cafazzo S, Abis A et al. Age-graded dominance hierarchies and social tolerance in packs of free-ranging dogs. Behav Ecol 2017; 28: 1004–20. 10.1093/beheco/arx059

[25] Cafazzo S , Lazzaroni M, Marshall-Pescini S. Dominance relationships in a family pack of captive arctic wolves (Canis lupus arctos): the influence of competition for food, age and sex. PeerJ 2016; 4: e2707. 10.7717/peerj.2707 27904806 PMC5126626

[26] Bailey I , Myatt JP, Wilson AM. Group hunting within the Carnivora: physiological, cognitive and environmental influences on strategy and cooperation. Behav Ecol Sociobiol 2013; 67: 1–17. 10.1007/s00265-012-1423-3

[27] Reynolds CW . Flocks, herds, and schools: a distributed behavioral model. Behav Ecol Sociobiol 1987; 21: 25–34. 10.1145/37401.37406

[28] Vicsek T , Czirók A, Ben-Jacob E et al. Novel type of phase transition in a system of self-driven particles. Phys Rev Lett 1995; 75: 1226–9. 10.1103/PhysRevLett.75.1226 10060237

[29] Couzin ID , Krause J, James R et al. Collective memory and spatial sorting in animal groups. J Theor Biol 2002; 218: 1–11. 10.1006/jtbi.2002.3065 12297066

[30] VáSárhelyi G , ViráGh C, Somorjai G et al. Optimized flocking of autonomous drones in confined environments. Sci Robot 2018; 3: eaat3536. 10.1126/scirobotics.aat3536 33141727

[31] Englebrecht AP . Fundamentals of Computational Swarm Intelligence. New Jersey: Wiley, 2005.

[32] Ilie S , Bădică C. Multi-agent distributed framework for swarm intelligence. Procedia Comput Sci 2013; 18: 611–20. 10.1016/j.procs.2013.05.225

[33] Saka MP , Doğan E, Aydogdu I. Analysis of swarm intelligence-based algorithms for constrained optimization. In: Yang XS, Cui ZH, Xiao RB et al., (eds). Swarm Intelligence and Bio-Inspired Computation. Amsterdam: Elsevier, 2013, 25–48. 10.1016/B978-0-12-405163-8.00002-8

[34] Garnier S , Gautrais J, Theraulaz G. The biological principles of swarm intelligence. Swarm Intell 2007; 1: 3–31. 10.1007/s11721-007-0004-y

[35] Beni G , Wang J. Swarm intelligence in cellular robotic systems. In: NATO Advanced Workshop on Robots and Biological Systems, Ciocco, Toscana, Italy. Berlin: Springer, 1989, 703–12.

[36] Millonas MM . Swarms, Phase Transitions, and Collective Intelligence. Boston: Addison Wesley, 1994.

[37] Zhang W , Mei H. A constructive model for collective intelligence. Natl Sci Rev 2020; 7: 1273–7. 10.1093/nsr/nwaa092 34692154 PMC8289164

[38] Bonabeau E , Dorigo M, Théraulaz G. Swarm Intelligence: From Natural to Artificial Systems. Oxford: Oxford University Press, 1999. 10.1093/oso/9780195131581.001.0001

[39] Kennedy J , Eberhart RC. Particle swarm optimization. In: IEEE International Conference on Neural Networks (ICNN), Perth, Australia. New York: IEEE Press, 1995, 1942–8. 10.1109/ICNN.1995.488968

[40] Dorigo M , Birattari M, Stützle T. Ant colony optimization. IEEE Comput Intell Mag 2006; 1: 28–39. 10.1109/MCI.2006.329691

[41] Çahin E . Swarm robotics: from sources of inspiration to domains of application. In: International Workshop on Swarm Robotics (SR), Santa Monica, CA, USA. Berlin: Springer, 2004, 10–20.

[42] Al-Obaidy M , Al-Azawi R. Cluster-based algorithm for energy optimization of swarmed robots using swarm intelligence. In: 2019 Sixth HCT Information Technology Trends (ITT), Ras Al Khaimah, United Arab Emirates. New York: IEEE Press, 2020, 202–7.

[43] Grassé PP . La reconstruction du nid et les coordinations interindividuelles chez*Bellicositermes natalensis* et*Cubitermes sp.* la théorie de la stigmergie: Essai d’interprétation du comportement des termites constructeurs. Insectes Soc 1959; 6: 41–80. 10.1007/BF02223791

[44] Trianni V , Nolfi S, Dorigo M. Evolution, self-organization and swarm robotics. Swarm Intell 2008; 6: 163–91. 10.1007/978-3-540-74089-6_5

[45] Tahira A , Böling J, Haghbayan MH et al. Swarms of unmanned aerial vehicles - a survey. J Ind Inf Integration 2019; 16: 100106. 10.1016/j.jii.2019.100106

[46] Cambone S . Unmanned Aircraft Systems Roadmap 2005-2030. Washington, DC: Office of the Secretary of Defense, 2005.

[47] China Electronics Standardization Institute . White Paper on the Development of Intelligent Unmanned Swarm Systems. http://www.cesi.cn/202111/8036.html (23 November 2021, date last accessed).

[48] China Electronics Standardization Institute . Information Technology-Unmanned Swarm-Terminology. http://www.ttbz.org.cn/StandardManage/Detail/62445/ (7 June 2022, date last accessed).

[49] CNET . See NASA test a swarm of 100 US Navy Cicada drones. https://www.cnet.com/science/see-nasa-test-a-swarm-of-100-us-navy-cicada-drones/ (22 April 2019, date last accessed).

[50] Air & Space Forces Magazine . C-130 catches an X-61 Gremlins vehicle in airborne recovery test. https://www.airforcemag.com/c-130-catches-x-61-gremlins-vehicle-airborne-recovery-test/ (5 November 2021, date last accessed).

[51] Kang YH , Luo DL, Xin B et al. Robust leaderless time-varying formation control for nonlinear unmanned aerial vehicle swarm system with communication delays. IEEE Trans Cybern 2022; doi: 10.1109/TCYB.2022.3165007. 10.1109/TCYB.2022.3165007 35580098

[52] Zhou SY , Dong XW, Tan QK et al. Time-varying group formation-tracking control for general linear multi-agent systems with switching topologies and unknown input. In: 22nd IEEE International Conference on Industrial Technology (ICIT), Valencia, Spain. New York: IEEE Press, 2021, 105–10.

[53] Huo MZ , Duan HB, Zeng ZG. Multi-cluster consensus for large-scale heterogenous manned/unmanned aerial team with random link failure via pinning control. IEEE Trans Circuits Syst II Express Briefs 2022; 69: 4924–8.

[54] Zhou LY , Zhao XT, Guan X et al. Robust trajectory planning for UAV communication systems in the presence of jammers. Chinese J Aeronaut 2022; 35: 265–74. 10.1016/j.cja.2021.10.038

[55] Tan ZW , Nguyen AHT, Khong AWH. An efficient dilated convolutional neural network for UAV noise reduction at low input SNR. In: Asia-Pacific Signal and Information Processing Association Annual Summit and Conference (APSIPA ASC), Lanzhou, China. New York: IEEE Press, 2019, 1885–92. 10.1109/APSIPAASC47483.2019.9023324

[56] Timothy HC , Michael RC, Michael AD et al. Live-fly, large-scale field experimentation for large numbers of fixed-wing UAVs. In: IEEE International Conference on Robotics and Automation (ICRA), Stockholm, Sweden. New York: IEEE Press, 2016, 1255–62.

[57] Huo MZ , Duan HB, Fan YM. Pigeon-inspired circular formation control for multi-UAV system with limited target information. Guid Navig Control 2021; 1: 2150004. 10.1142/S2737480721500047

[58] Zhang DF , Duan HB, Fan YM et al. UAV swarm containment control inspired by spatial interaction mechanism of wolf-pack foraging. Sci Sin Technol 2022; 52: 1555–70. 10.1360/SST-2021-0042

[59] Duan HB , Zhang DF, Fan YM et al. From wolf pack intelligence to UAV swarm cooperative decision-making. Sci Sin Inform 2019; 49: 112–8. 10.1360/N112018-00168

[60] Duan HB , Zhang DF. A binary tree based coordination scheme for target enclosing with micro aerial vehicles. IEEE/ASME Trans Mechatron 2021; 26: 458–68.

[61] Yu X , Xu X, Liu L et al. Circular formation of networked dynamic unicycles by a distributed dynamic control law. Automatica 2018; 89: 1–7. 10.1016/j.automatica.2017.11.021

[62] Yao WR , Chen Y, Tian HY et al. Multi-UAV synchronous approaching using homotopy-based trajectory planning. Guid Navig Control 2022; 2: 2250012. 10.1142/S2737480722500121

[63] Feng Q , Hai XS, Sun B et al. Resilience optimization for multi-UAV formation reconfiguration via enhanced pigeon-inspired optimization. Chinese J Aeronaut 2022; 35: 110–23. 10.1016/j.cja.2020.10.029

[64] Liao F , Teo R, Wang JL et al. Distributed formation and reconfiguration control of VTOL UAVs. IEEE Trans Control Syst Technol 2017; 25: 270–7. 10.1109/TCST.2016.2547952

[65] Chen L , Duan HB. Collision-free formation-containment control for a group of UAVs with unknown disturbances. Aerosp Sci Technol 2022; 126: 107618. 10.1016/j.ast.2022.107618

[66] Yuan GS , Xia J, Duan HB. A continuous modeling method via improved pigeon-inspired optimization for wake vortices in UAVs close formation flight. Aerosp Sci Technol 2022; 120: 107259. 10.1016/j.ast.2021.107259

[67] Vásárhelyi G , Virágh C, Somorjai G et al. Optimized flocking of autonomous drones in confined environments. Sci Robot 2018; 3: eaat3536. 10.1126/scirobotics.aat3536 33141727

[68] Zhou X , Wen XY, Wang ZP et al. Swarm of micro flying robots in the wild. Sci Robot 2022; 7: eaat5954 10.1126/scirobotics.abm5954 35507682

[69] Global Times . China unveils first practical drone swarm tech, ‘to be used in amphibious landing missions’. https://www.globaltimes.cn/page/202010/1203857.shtml (18 October 2020, date last accessed).

[70] U.S. Army . Interoperability key to successful EDGE22, future conflicts. https://www.army.mil/article/256737/interoperability-key-to-successful-edge22-future-conflicts (16 May 2022, date last accessed).

[71] Xie H , Sun MM an Fan XJ et al. Reconfigurable magnetic microrobot swarm: multimode transformation, locomotion, and manipulation. Sci Robot 2019; 4: eaav8006. 10.1126/scirobotics.aav8006 33137748

[72] Yang LD , Jiang Jl, Gao XJ et al. Autonomous environment-adaptive microrobot swarm navigation enabled by deep learning-based real-time distribution planning. Nat Mach Intell 2022; 4: 480–93. 10.1038/s42256-022-00482-8

[73] Tao B , Zhao XW, Ding H. Study on robotic mobile machining techniques for large complex components. Sci China Technol Sci 2018; 48: 1302–12. 10.1360/N092018-00192

[74] Sato N , Wakabayashi Y. JEMRMS design features and topics from testing. In: 6th International Symposium on Artificial Intelligence and Robotics & Automation in Space (SAIRAS), Quebec, Canada. Paris: European Space Agency, 2001, 1–7.

[75] Zhou JP . Chinese space station project overall vision. Manned Spaceflight 2013; 19: 1–10.

[76] Chen J , Kai SX. Cooperative transportation control of multiple mobile manipulators through distributed optimization. Sci China Inf Sci 2018; 61: 120201. 10.1007/s11432-018-9588-0

[77] Galicki M . Control of mobile manipulators in a task space. IEEE Trans Automat Contr 2012; 57: 2962–7. 10.1109/TAC.2012.2195935

[78] Rodríguez-Araújo J , Rodríguez-Andina JJ, Fariña J et al. Field-programmable system-on-chip for localization of UGVs in an indoor iSpace. IEEE Trans Industr Inform 2014; 10: 1033–43. 10.1109/TII.2013.2294112

[79] Qin HL , Meng ZH, Meng W et al. Autonomous exploration and mapping system using heterogeneous UAVs and UGVs in GPS-denied environments. IEEE Trans Veh Technol 2019; 68: 1339–50. 10.1109/TVT.2018.2890416

[80] Chen J , Zhang X, Xin B et al. Coordination between unmanned aerial and ground vehicles: a taxonomy and optimization perspective. IEEE Trans Cybern 2016; 46: 959–72. 10.1109/TCYB.2015.2418337 25898328

[81] Pitonakova L , Winfield A, Crowder R. Recruitment near worksites facilitates robustness of foraging E-puck swarms to global positioning noise. In: IEEE/RSJ International Conference on Intelligent Robots and Systems (IROS), Madrid, Spain. New York: IEEE Press, 2018, 4276–81. 10.1109/IROS.2018.8593788

[82] Rubenstein M , Cornejo A, Nagpal R. Programmable self-assembly in a thousand-robot swarm. Science 2014; 345: 795–9. 10.1126/science.1254295 25124435

[83] Li SG , Batra R, Brown D et al. Particle robotics based on statistical mechanics of loosely coupled components. Nature 2019; 567: 361–5. 10.1038/s41586-019-1022-9 30894722

[84] IEEE Spectrum . Boston dynamics enters warehouse robots market, acquires kinema systems. https://spectrum.ieee.org/boston-dynamics-warehouse-robots-acquires-kinema-systems#toggle-gdpr (2 April 2019, date last accessed).

[85] Nguyen LA , Harman TL, Fairchild C. Swarmathon: a swarm robotics experiment for future space exploration. In: IEEE International Symposium on Measurement and Control in Robotics (ISMCR), Houston, TX, USA. New York: IEEE Press, 2019, 1–4. 10.1109/ISMCR47492.2019.8955661

[86] Poster R , Huczala D, Vysocký A et al. Modular rover design for exploration and analytical tasks. In: International Conference on Modelling and Simulation for Autonomous Systems, Palermo, Italy. Cham: Springer, 2019, 203–15.

[87] AUVSI . DARPA’S OFFSET program deploys swarms of autonomous air and ground vehicels during third field experiment. https://www.auvsi.org/industry-news/darpas-offset-program-deploys-swarms-autonomous-air-and-ground-vehicles-during-third (30 January 2020, date last accessed).

[88] Sahoo A , Dwivedy SK, Robi PS. Advancements in the field of autonomous underwater vehicle. Ocean Eng 2019; 181: 145–60. 10.1016/j.oceaneng.2019.04.011

[89] Xin B , Zhang JX, Chen J et al. Overview of research on transformation of multi-AUV formations. Complex Syst Mod Sim 2021; 1: 1–14. 10.23919/CSMS.2021.0003

[90] Elege N , Solapurkar S, Joordens M. Eye sensor for swarm robotic fish. In: 12th System of Systems Engineering Conference (SoSE), Waikoloa, HI, USA. New York: IEEE Press, 2017, 1–6. 10.1109/SYSOSE.2017.7994956

[91] Anguita D , Brizzolara D, Ghio A et al. Smart plankton: a nature inspired underwater wireless sensor network. In: 4th International Conference on Natural Computation, Jinan, China. New York: IEEE Press, 2008, 701–5. 10.1109/ICNC.2008.634

[92] Szeliski R . Computer Vision: Algorithms and Applications. Berlin: Springer, 2010.

[93] Yan ZP , Zhang C, Tian WD et al. Formation trajectory tracking control of discrete-time multi-AUV in a weak communication environment. Ocean Eng 2022; 245: 110495. 10.1016/j.oceaneng.2021.110495

[94] Suryendu C , Subudhi B. Formation control of multiple autonomous underwater vehicles under communication delays. IEEE Trans Circuits Syst II Express Briefs 2020; 67: 3182–6. 10.1109/TCSII.2020.2976955

[95] Wei HL , Shen C, Shi Y. Distributed Lyapunov-based model predictive formation tracking control for autonomous underwater vehicles subject to disturbances. IEEE Trans Syst Man Cybern Syst 2019; 51: 5198–208. 10.1109/TSMC.2019.2946127

[96] Wang YQ , Hu RY, Huang SH et al. Passive inverted ultra-short baseline positioning for a disc-shaped autonomous underwater vehicle: design and field experiments. IEEE Robot Autom Lett 2022; 7: 6942–9. 10.1109/LRA.2022.3178494

[97] Yan ZP , Zhang MY, Zhang C et al. Decentralized formation trajectory tracking control of multi-AUV system with actuator saturation. Ocean Eng 2022; 255: 111423. 10.1016/j.oceaneng.2022.111423

[98] Liu H , Wang YH, Lewis FL. Robust distributed formation controller design for a group of unmanned underwater vehicles. IEEE Trans Syst Man Cybern 2021; 51: 1215–23. 10.1109/TSMC.2019.2895499

[99] Yuan CZ , Licht S, He HB. Formation learning control of aultiple autonomous underwater vehicles with heterogeneous nonlinear uncertain dynamics. IEEE Trans Cybern 2018; 48: 2920–34. 10.1109/TCYB.2017.2752458 28961137

[100] Berlinger F , Gauci M, Nagpal R. Implicit coordination for 3D underwater collective behaviors in a fish-inspired robot swarm. Sci Robot 2021; 6: eabd8668. 10.1126/scirobotics.abd8668 34043581

[101] Yu JZ , Wang C, Xie GM. Coordination of multiple robotic fish with applications to underwater robot competition. IEEE Trans Ind Electron 2016; 63: 1280–8. 10.1109/TIE.2015.2425359

[102] Schulz B , Hobson B, Kemp M et al. Field results of multi-UUV missions using ranger micro-UUVs. In: Oceans 2003. Celebrating the Past ... Teaming Toward the Future (IEEE Cat. No.03CH37492), San Diego, CA, USA. New York: IEEE Press, 2003, 956–61. 10.1109/OCEANS.2003.178457

[103] Tether T . Statement of the director of the Defense Advanced Research Projects Agency submitted to the Subcommittee on Terrorism, Unconventional Threats and Capabilities, House Armed Services Committee, United States House of Representatives, 2008.

[104] Wang H , Li JJ, Cai L et al. Flight control of robo-pigeon using a neural stimulation algorithm. J Integr Neurosci 2018; 17: 337–42.

[105] Wang H , Wu J, Fang K et al. Application of robo-pigeon in ethological studies of bird flocks. J Integr Neurosci 2020; 19: 443–8. 10.31083/j.jin.2020.03.159 33070523

[106] Papadopoulou M , Hildenbrandt H, Sankey DWE et al. Self-organization of collective escape in pigeon flocks. PLoS Comput Biol 2022; 18: e1009772. 10.1371/journal.pcbi.1009772 35007287 PMC8782486

[107] Schwerd S , Schulte A. Operator state estimation to enable adaptive assistance in manned-unmanned-teaming. Cogn Syst Res 2021; 67: 73–83. 10.1016/j.cogsys.2021.01.002

[108] Roth G , Schulte A, Schmitt F et al. Transparency for a workload-adaptive cognitive agent in a manned-unmanned teaming application. IEEE Trans Hum Mach Syst 2020; 50: 225–33. 10.1109/THMS.2019.2914667

[109] Chen J , Xin B. Key scientific problems in the autonomous cooperation of manned-unmanned systems. Sci Sin Inform 2018; 48: 1270–4. 10.1360/N112018-00092

[110] Zhang FB , Yu J, Lin DF et al. UnIC: towards unmanned intelligent cluster and its integration into society. Engineering 2022; 12: 24–38. 10.1016/j.eng.2022.02.008

[111] Kolling A , Walker P, Chakraborty N et al. Human interaction with robot swarms: a survey. IEEE Trans Hum Mach Syst 2016; 46: 9–26. 10.1109/THMS.2015.2480801

[112] Kolling A Sycara K , Nunnally S et al. Human swarm interaction: an experimental study of two types of interaction with foraging swarms. Int J Rob Res 2013; 2: 103–28.

[113] Kira Z , Potter M. Exerting human control over decentralized robot swarms. In: 4th International Conference on Autonomous Robots and Agents, Wellington, New Zealand. New York: IEEE Press, 2009, 566–71. 10.1109/ICARA.2000.4803934

[114] Walter B , Sannier A, Reiners D et al. UAV swarm control: calculating digital pheromone fields with the GPU. J Def Model Simul 2006; 3: 167–76. 10.1177/154851290600300304

[115] Walker P , Amraii SA, Lewis M et al. Human control of leader-based swarms. In: IEEE International Conference on Systems, Man, and Cybernetics, Manchester, UK. New York: IEEE Press, 2013, 2712–7. 10.1109/SMC.2013.462

[116] Nam C , Walker P, Li H et al. Models of trust in human control of swarms with varied levels of autonomy. IEEE Trans Hum Mach Syst 2020; 50: 194–204. 10.1109/THMS.2019.2896845

[117] Niu YF , Shen LC, Li J et al. Key scientific problems in cooperation control of unmanned-manned aircraft systems. Sci Sin Inform 2019; 49: 538–54. 10.1360/N112019-00008

[118] Zhou ZY , Liu JC, Yu JZ. A survey of underwater multi-robot systems. IEEE/CAA J Autom Sinica 2022; 9: 1–18. 10.1109/JAS.2021.1004269

[119] Liu H , Li ZQ, Liu YW et al. Key technologies of TianGong-2 robotic hand and its on-orbit experiments. Sci Sin Technol 2018; 48: 1313–20. 10.1360/N092018-00168

[120] Duan HB , Xu XB. Create machine vision inspired by eagle eye. Research 2022; 2022: 9891728. 10.34133/2022/9891728 PMC11412415 39301503

[121] Duan HB , Huo MZ. Pigeon-Inspired Optimization. Beijing: Science Press, 2023.

